# Utility of cardiac implantable electronic device algorithm for detecting severe sleep‐disordered breathing in cardiomyopathy

**DOI:** 10.1002/joa3.13156

**Published:** 2024-10-08

**Authors:** Jiaqi Li, Yingjuan Mok, Vern Hsen Tan, Hang Siang Wong, Yue Wang, Ying Zi Oh, Ai Ling Him, Sherida Syed Hamid, Prunella Ting Lee, Lisa Jie Ting Teo, Leng Leng Lee, Andrew Kieran Ming Hui Chan, Colin Yeo

**Affiliations:** ^1^ School of Clinical Medicine University of Cambridge Cambridge UK; ^2^ Department of Sleep Medicine, Surgery & Science Changi General Hospital Singapore Singapore; ^3^ Department of Respiratory Medicine Changi General Hospital Singapore Singapore; ^4^ Department of Cardiology Changi General Hospital Singapore Singapore; ^5^ Clinical Measurement Unit Changi General Hospital Singapore Singapore

**Keywords:** CIED, heart failure, ICD, sleep‐disordered breathing

## Abstract

**Background:**

Half of patients with heart failure are estimated to have sleep‐disordered breathing (SDB). However, many are undiagnosed as they do not report typical symptoms. This study aims to evaluate the implantable cardiac defibrillator (ICD) sleep‐disordered breathing algorithm in a cohort of multi‐racial Asian patients for detection of SDB against polysomnography (PSG).

**Methods:**

In this prospective pilot study, participants who fulfill the American College of Cardiology (ACC) indication for ICD were recruited. The ICD algorithm uses transthoracic impedance sensing to calculate respiratory disturbance index (RDI).

**Results:**

Twenty‐four patients were enrolled between August 2020 and December 2021. All patients underwent PSG exams and were followed up for up to 12 months. Eighteen participants completed the PSG study as of August 23, 2022. Severe SDB (defined as PSG‐AHI ≥30 episodes/h) was diagnosed in 66.7% of the patients. No significant direct linear correlation was found between the PSG‐AHI measurements and the RDI measurements (adjusted *r* ^2^ = .224, *r* = .473, *p* = .027). Applying a binary threshold cut‐off RDI value of 32 episodes/h for the detection of severe SDB yielded a sensitivity of 91.7% and specificity of 16.7%.

**Conclusions:**

Transthoracic impedance sensing with an advanced inbuilt algorithm may be helpful as a screening test in detecting severe SDB in patients with heart failure and cardiomyopathy, potentially by applying a binary threshold cut‐off value. This is the first study known to validate the algorithm in an exclusively multi‐ethnic Asian population with heart failure.

## Graphical abstract

ICD‐transthoracic impedance algorithm in screening for severe sleep apnea. Our study evaluated the ApScan® algorithm in implantable cardiac defibrillators (ICD) to screen for severe sleep apnea in a multi‐ethnic Asian cohort with heart failure. Applying a binary threshold cut‐off may be useful in detecting severe sleep apnea with high specificity in this group of ICD patients.

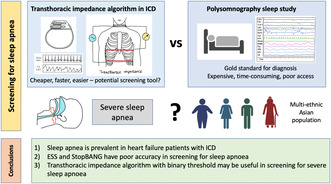

## 1 INTRODUCTION

Sleep‐disordered breathing (SDB) is highly prevalent in patients with cardiovascular diseases including heart failure^1^ and is a marker of poor clinical outcomes. It is estimated that 10% of patients over the age of 70 suffer from heart failure,^2^ and up to 80% of patients with heart failure may have SDB.^3^ Thus, SDB confers significant disease burden as well as high morbidity.^4^

Unfortunately, many patients with heart failure remain undiagnosed and untreated, as they often do not report typical symptoms of SDB. Furthermore, the diagnosis of SDB in these patients usually requires the gold standard, in‐laboratory polysomnography (PSG), an investigation that is expensive and time‐consuming, and access may be limited.

Existing screening questionnaires are used in primary care to evaluate the risk of SDB. These include the Epworth Sleepiness Scale (ESS) and the STOP‐Bang questionnaires. However, previous studies have shown these questionnaires to have poor sensitivity in patients with heart failure,^5^ as this cohort of patients have less subjective daytime sleepiness compared to those without heart failure, despite significantly reduced sleep time.^6^ Given the practical and cost limitations of PSG and the poor sensitivity of existing screening questionnaires, there is a need for the development of further screening tools that are more effective in this group of patients.

Recent models of cardiac implantable electronic devices (CIED) incorporate an SDB scan function, which have shown promising results. These devices measure thoracic impedance changes, which can be used to detect respiratory events, as transthoracic impedance is increased during inspiration and decreased during expiration. Promising results have been shown in pacemaker (PPM) patient cohorts: data from a European cohort using the Sorin pacemaker (PPM) and a Chinese cohort with the Boston Scientific PPM suggested that this may be useful in screening for SDB.^1^, ^7^

However, few studies have evaluated the performance of such transthoracic impedance algorithms in patients with implantable cardiac defibrillators (ICD), and existing studies show mixed results. The DASAP‐HF study suggested that the Boston Scientific ApScan® algorithm may be effective in screening for patients at risk of SDB.^8^ However, a separate study from the UPGRADE trial looking at a similar cohort and the same algorithm suggested poor sensitivity in detecting SDB and hence limited utility.^9^

Given the limited number of studies and the conflicting data regarding ApScan® in detecting SDB within an ICD cohort, further research is warranted. Moreover, both the DASAP‐HF and UPGRADE studies were exclusively made up of European populations. Similarly, a recent meta‐analysis assessing the utility of all implantable cardiac devices (PPM and ICDs) revealed that among the 16 included studies, only 4 examined patients with implanted ICDs, all within Caucasian populations.^10^ Hence, our study seeks to prospectively investigate the efficacy of the ApScan® algorithm in a multi‐racial Asian population to ascertain its effectiveness across broader demographics (multi‐racial Asian ICD patient cohort). Ultimately, development and improvement of a device‐based screening system can better identify patients who could benefit from early referral for diagnosis and treatment of severe SDB.

Using the ApScan® algorithm, we aim to validate its sensitivity and specificity against the gold standard polysomnography (PSG). Additionally, we also compared the performance of the ApScan® algorithm with existing ESS and STOP‐Bang questionnaires. This comprehensive comparison allows evaluation of gold‐standard diagnosis and existing screening tools to provide insight into the algorithm's utility in diverse patient populations.

## 2 MATERIALS AND METHODS

### 2.1 Study design

This prospective single‐center study was conducted in Changi General Hospital, Singapore.

Inclusion criteria were all patients who fulfilled the American College of Cardiology (ACC) and European Society of Cardiology (ESC) indication for implantable cardiac defibrillator (ICD) of primary or secondary prevention of sudden cardiac death and agreed to be on a remote monitor. Informed consent was obtained from participants. Participants with a known diagnosis of sdb, who declined remote monitor or a life expectancy of less than 1 year were excluded. A total of twenty‐four patients were included in the study. Eighteen participants completed the study.

Participants received Boston Scientific Charisma ICDs and underwent remote monitoring. All the patients ICD base rate was programmed as 40 beats per minute unless explicitly requested otherwise by implanting physicians. All our patients' ICD base rate was programmed at 40 beats per minute on implant. The ICD implantation procedures were conducted between August 27, 2020 and December 16, 2021, with all devices implanted in the right ventricle (RV) tip position at the apical septum.

Information including demographic characteristics, underlying cardiovascular and respiratory disease, cardiovascular medications and GDMT, and pacing indications were collected. Patients also undertook health and screening questionnaires: the 36‐item Short Form survey (SF‐36), Epworth Sleepiness Scale (ESS), and STOP‐Bang questionnaire (see Supplementary Tables S2 and S3).

The trial was investigator‐initiated. The research group comprising the authors was responsible for design, execution and conduct of the study. All members of the group approved the statistical analyses and interpretation of the data. The decision to publish the results and the decisions regarding the contents of the manuscript were made by the group. The authors attest to the accuracy of the data and of all the analyses and to the fidelity of the report. The study was sponsored by Boston Scientific via an unrestricted grant. The sponsor was not involved in the design, evaluation of results, or writing of the manuscript.

### 2.2 Patient follow‐up

Participants were followed up for 1‐year post‐ICD implantation for clinical events including any hospitalizations, acute coronary syndrome (ACS) events, death and cardiovascular death, and the number of shocks delivered by devices.

**FIGURE 1:**
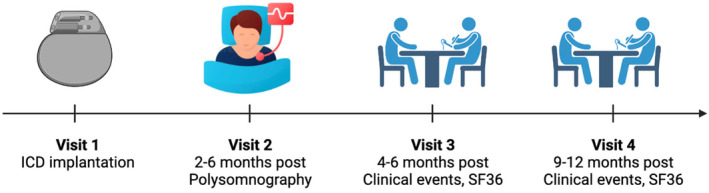
Data collection timeline. Schematic of data collection timepoints after ICD implantation. Figure created with illustrations from BioRender.com.

All patients had 4 visits in total (Figure 1). Visit 1 represents the date of ICD implantation. Visit 2 was the date of the sleep study (polysomnography, PSG). Respiratory disturbance index (RDI) data were also collected on the index night of the PSG study. RDI data collection was commenced at least 2 months after the ICD implant to avoid any fluctuations of impedance in the first 2 months as a result of a newly implanted ICD lead. Visits 3 and 4 were carried out at timepoints of 4–6 months and 9–12 months post‐ICD implantation, respectively, to monitor for any clinical events and repeat the SF‐36 quality‐of‐life questionnaire.

### 2.3 SDB screening questionnaires: Epworth sleepiness scale and STOP‐Bang

All participants took the Epworth Sleepiness Scale (ESS) and STOP‐Bang questionnaires. ESS is a subjective questionnaire where participants rate their chances of sleeping in eight situations on a 4‐point scale with a minimum score of 4 and a maximum score of 24. ESS of >10 warrants further medical assessment for SDB.^11^ The STOP‐Bang questionnaire is used to screen for SDB and incorporates subjective feelings of sleepiness and objective measurements including BMI, age, neck circumference, and gender.^12^ Sensitivity, specificity, positive predictive value (PPV), and negative predictive value (NPV) of the 2 questionnaires for severe SDB were determined.

### 2.4 Polysomnography (PSG) measurements

The participants underwent in‐laboratory polysomnography (PSG) for evaluation of sleep‐disordered breathing. PSG examination included electroencephalogram (EEG) derivatives, electrooculography, oronasal pressure cannula, thermistor, thoracic and abdominal respiratory movement belts, pulse oximetry, submental, and anterior tibialis electromyogram (EMG). PSG‐RDI data were also collected on the index night of the PSG study. The PSGs were scored in accordance with the American Academy of Sleep Medicine 2012 standard criteria. Parameters and data collected from the PSG study are detailed in Supplementary Table S1. The apnea‐hypopnea index (AHI) was used to grade the severity of SDB using standard cut‐offs as per the International Classification of Sleep Disorders (ICSD): mild SDB: 5–14 episodes/h; moderate SDB 15–29 episodes/h; severe ≥30 episodes/h.^13^

### 2.5 Transthoracic impedance and ApScan® algorithm

We utilized the Boston Scientific ICD, equipped with an integrated Apnoea Scan (ApScan®) algorithm that employs transthoracic impedance sensing to monitor sleep‐disordered breathing, quantified as a respiratory disturbance index (RDI). Cardiac physiologists collected RDI data remotely from the monitor on the day of the polysomnogram (PSG). A comparison was drawn between the AHI obtained from the PSG study (PSG‐AHI) and the RDI recorded on the index night. We had utilized a uniform monitoring window of 10:00 p.m. to 4:00 a.m., with RDI measurements specifically recorded between 11:00 p.m. and 3:00 a.m. This consistent measurement timeframe was used for all patients in the study. Notably, no invalid measurements were made by ApScan® during the index night.

### 2.6 Statistical analysis

Using the inbuilt ApScan® algorithm in the ICD, we evaluate the utility against gold‐standard PSG. Categorical variables were presented as numbers and percentages. Continuous variables were presented as median ± IQR. Agreement between the two methods (RDI and PSG‐AHI) was analyzed using Bland–Altman statistics. Pearson's correlation coefficient was used to evaluate the direct relationship between RDI and PSG‐AHI.

An optimal cut‐off value was applied to maximize the sensitivity and specificity of RDI in predicting severe SDB. Sensitivity, specificity, positive predictive value (PPV), and negative predictive value (NPV) were calculated. All statistical analyses were performed by R 4.2.1 software. A *p* value of <.05 was considered statistically significant.

### 2.7 Ethical approval

Institution ethics committee approval was obtained for this study (Singhealth Ethics approval 2020/2141).

## 3 RESULTS

### 3.1 Study population

Twenty‐four patients were enrolled and underwent ICD implantation from August 27, 2020 to December 16, 2021, at Changi General Hospital, Singapore. Eighteen participants completed the PSG study as of August 23, 2022. Median age of participants was 65.0 years (IQR 60.25–70.75) with BMI 26.1 (23/43–27.82). 16/18 (88.9%) were male, and 11/18 (61.1%) were Chinese, 2/18 (11.1%) were Malay, 3/18 (16.7%) were Indian, and 2/18 (11.1%) were others, which generally is representative of the background demographics in Singapore.

Data on heart failure medications, specifically Goal‐Directed Medical Therapy (GDMT), were collected. 16/18 (88.9%) of patients were on a beta blocker, 15/18 (83.3%) of patients were on RAAS blockade agents of either ACE‐I/ARB or an ARNI, 10/18 (55.6%) of patients were on a mineralocorticoid receptor antagonist, and 12/18 (66.7%) were on a SGLT2 inhibitor.

12/18 (66.7%) of patients had ICD implanted for primary prevention. Etiology of cardiomyopathy was ischemic in 15/18 (83.3%) and due to channelopathy in 1 (0.55%) of patients. The patient characteristics are shown in Table 1.

**TABLE 1:** Demographic and clinical characteristics.

Characteristics	Total (*n* = 18)
Male	16 (88.9%)
Race	
Chinese	11 (61.1%)
Malay	2 (11.1%)
Indian	3 (16.7%)
Others	2 (11.1%)
Age (year)	Mean ± SD: 65.6 ± 7.92 Median IQR: 65.0 (60.25–70.75)
BMI (kg/m^2^)	Mean ± SD: 26.5 ± 4.08 Median IQR: 26.1 (23.43–27.82)
Final diagnosis	
Obstructive sleep apnea	15 (83.3%)
Central sleep apnea	1 (5.6%)
Mixed sleep apnea	2 (11.1%)
Smoker	2 (11.1%)
Ex‐smoker	7 (38.9%)
ICD indication	
Primary prevention	12 (66.7%)
Secondary prevention	6 (33.3%)
Comorbidities	
Diabetes	13 (72.2%)
IHD	14 (77.8%)
Hypertension	16 (88.9%)
Stroke/TIA	1 (0.55%)
PVD	0 (0.00%)
AF	2 (11.1%)
COPD	0 (0.00%)
Asthma	2 (11.1%)
Etiology of cardiomyopathy	
Ischemic	15 (83.3%)
Channelopathy	1 (0.55%)
LVEF	
>50%	2 (11.1%)
40%–49%	0 (0%)
30%–39%	3 (16.7%)
<30%	13 (72.2%)
ESS score ≥11	3 (16.7%)
STOPBANG score ≥3	17 (94.4%)
PSG‐AHI (episodes/h)	Mean ± SD: 41.3 ± 19.18 Median IQR: 44.4 (23.95–57.85)
ICD‐RDI (episodes/h)	Mean ± SD: 42.3 ± 10.96 Median IQR: 40.0 (34.50–49.00)

### 3.2 Prevalence of sleep‐disordered breathing

The median PSG‐AHI recorded was 44.4 (IQR 23.95–57.85) episodes/h on the index night. The prevalence of SDB was 100% in our patients (11.1% mild, 22.2% moderate, and 66.7% severe SDB). 16.7% of patients had mixed obstructive and central SDB (Table 1).

### 3.3 Primary outcome: Utility of RDI in detecting SDB

**FIGURE 2:**
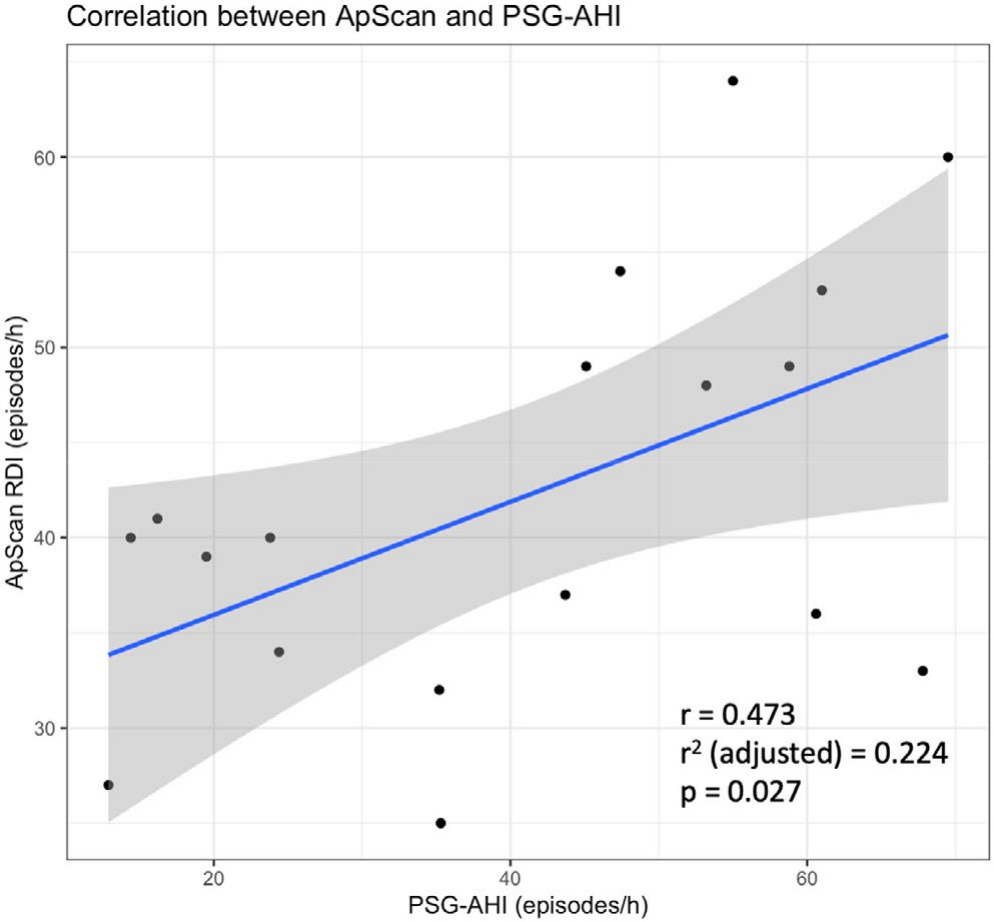
Linear correlation between ApScan® RDI and PSG‐AHI. Scatter plot of ApScan® RDI measurements and PSG‐AHI on the index night.

**FIGURE 3:**
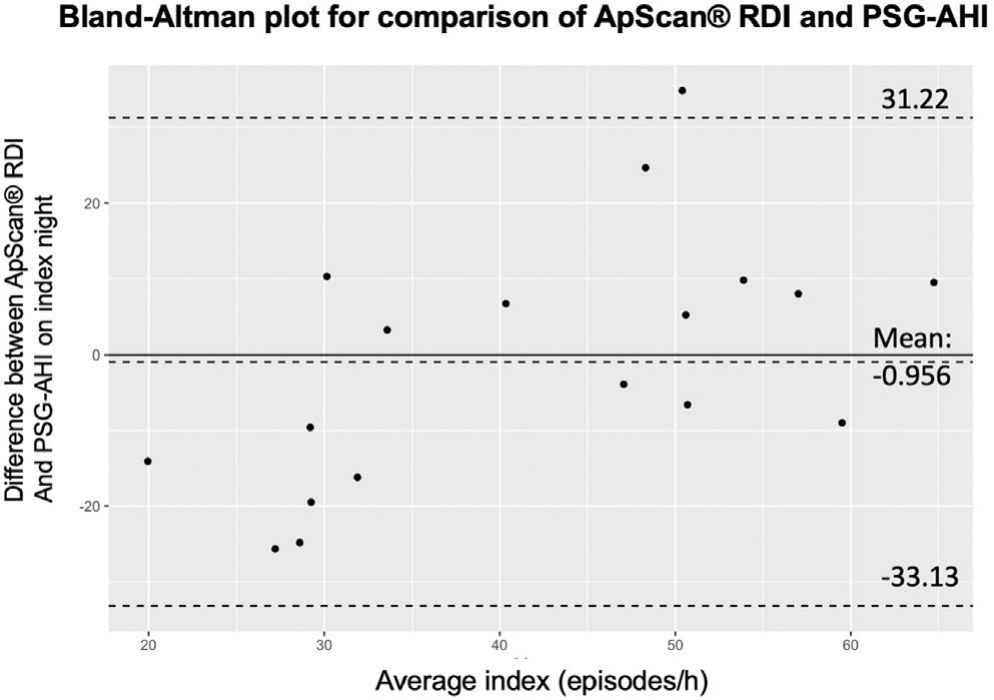
Bland–Altman plot of PSG‐AHI and ApScan® RDI. *y*‐axis: Difference between PSG‐AHI and ApScan® RDI measurements in the index night; *x*‐axis: Average index of PSG‐AHI and ApScan® RDI on index night. The mean of the differences (bias) is shown with a 95% confidence interval. The solid line indicates the bias, and dashed lines represent limits of agreement between indices. PSG‐AHI, polysomnography apnea‐hypopnea index; RDI, respiratory disturbance index.

Severe SDB (defined as PSG‐AHI ≥30 episodes/h) was diagnosed by PSG in 66.7% of the patients. RDI was found to have a positive correlation with PSG‐AHI (*r* = .473, *p* = .027) (Figure 2). The degree of bias is demonstrated by the Bland–Altman method (Figure 3), with the mean discrepancy of −0.956, 95% CI −33.13 to 31.22.

### 3.4 Sensitivity and specificity of ApScan® RDI in detecting severe sleep‐disordered breathing by PSG‐AHI analysis

**FIGURE 4:**
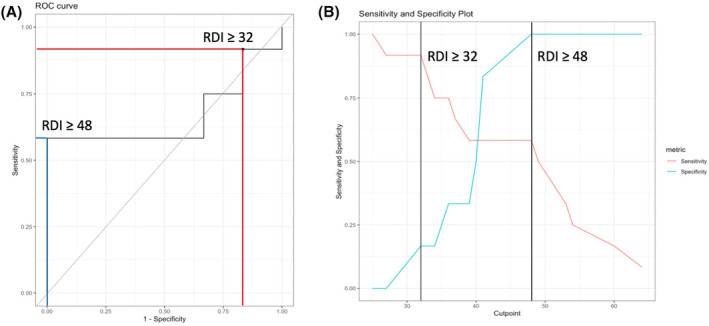
Receiver operating characteristic (ROC) curve analysis of RDI. (A) ROC curve and (B) sensitivity and specificity plot. Manufacturer's recommended threshold of RDI ≥32 for severe sleep apnea and ROC metric‐optimized threshold of RDI ≥48 are shown. RDI, respiratory disturbance index.

Using the cut‐off threshold of RDI for severe SDB of 32 (as per manufacturer's suggestions), this produces a sensitivity of 91.7% and a specificity of 16.7%. It is also worthwhile to note that a cut‐off value of 48 produces a sensitivity of 58.3% and a specificity of 100% (Figure 4).

### 3.5 Secondary outcomes

The utility of the Epworth Sleepiness Scale and STOP‐Bang questionnaires was determined. ESS had a poor sensitivity of 17.6% and specificity of 100% (PPV = 100%, NPV = 6.7%); STOP‐Bang had a high sensitivity of 94.1% but poor specificity of 0% (PPV = 94.1%, NPV = 0%) in diagnosing SDB.

### 3.6 Long‐term follow‐up

Two patients were admitted for heart failure during the course of monitoring 1‐year post‐ICD implantation, of which 1 patient was admitted 8 times. Five patients had documented acute coronary syndrome. No shocks were delivered to the 18 patients during the follow‐up period.

## 4 DISCUSSION

### 4.1 Main findings

In our multi‐racial Asian population requiring ICD implantation, 66.7% of patients had severe SDB. Using the cut‐off threshold of RDI for screening of severe SDB of 32 (as per the manufacturer's suggestions), this produces a sensitivity of 91.7% and a specificity of 16.7%. RDI of 48 episodes/h demonstrated a specificity of 100% and a sensitivity of 58.3% for severe SDB.

### 4.2 Prevalence of SDB in ICD patients

In our multi‐racial Asian cohort requiring ICD implantation, the prevalence of SDB was 100% (18/18), of which 66.7% had severe SDB. This reflects the high prevalence of SDB in this cohort of patients with heart failure. Reports indicate that the prevalence of SDB in populations with CIEDs varies significantly, ranging from 22% to 91%.^10^ However, this prevalence encompasses not only individuals with heart failure necessitating an ICD, as per our patient population, but also those with other indications for a PPM. It is established that individuals with heart failure tend to exhibit a higher incidence of SDB. It was also previously reported that Asians, despite being non obese, have more severe RDI. This is postulated to be due to differences in their craniofacial anatomy compared to Caucasian counterparts. (Kasey et al. The Laryngoscope 2020).

In this pilot study, we included patients with obstructive sleep apnea (OSA), central sleep apnea (CSA), and mixed sleep apnea (MSA). The distribution of our small cohort consisted of 15 patients with OSA, 1 with CSA, and 2 with MSA. We chose not to exclude CSA or MSA patients despite the different underlying mechanisms, as our primary aim was to gather preliminary data and assess the feasibility of the study across a spectrum of sleep apnea types.

### 4.3 Utility of ApScan® RDI in ICD patients

While previous studies utilizing transthoracic impedance algorithms in pacemakers have shown promising results in detecting SDB,^1^, ^7^ research within the ICD patient cohort, specifically among the non‐Caucasian population, remains limited. Among the existing studies, findings have also been inconclusive, with the DASAP‐HF study demonstrating good correlation (same‐day RDI and PSG‐AHI *r* = .74),^8^ while a separate study reported poor correlation (*r =* .41).^9^

In our study, conducted among a multi‐racial Asian cohort with cardiomyopathy, ApScan® RDI measurements did not exhibit a significant direct linear correlation with PSG‐AHI (*r* = .473, adjusted *r* ^2^ = .224, *p* = .027) (Figure 2). However, it is important to note that this correlation encompasses patients with all degrees of SDB, including mild, moderate, and severe cases. Therefore, we applied a binary threshold cut‐off to assess the utility of RDI in distinguishing between severe and nonsevere SDB (including no SDB, mild–moderate SDB).

When applying an RDI threshold cut‐off of 48 episodes/h for severe SDB, we observed a sensitivity of 58.3 and specificity of 100%. Of note, the manufacturer's suggested RDI threshold cut‐off for severe SDB is 32. When applied to our data, this threshold yielded high sensitivity but low specificity (Table 2). Conversely, the threshold of 48 optimized specificity but resulted in a lower sensitivity of 58.3%.

**TABLE 2:** Comparison of the sensitivity and specificity of screening tools used in our multi‐racial Asian population with ICD implantation for cardiomyopathy.

Screening tool	Sensitivity (%)	Specificity (%)
ApScan® *Cut‐off threshold of 32 for severe SA*	91.7	16.7
ApScan® *Cut‐off threshold of 48 for severe SA*	58.3	100
ESS questionnaire	17.6	100
STOP‐Bang questionnaire	94.1	0

Screening tests prioritize sensitivity; hence, the recommended cut‐off threshold of 32 episodes/h is useful as a screening test to detect severe SDB. Individuals identified should then be referred for PSG for diagnosis. We hypothesize that the difference in thresholds may stem from the algorithm being developed primarily within a Caucasian population, with the value of 32 optimized for that population. Differences in transthoracic impedance among predominantly Asian populations, potentially attributable to variances in thoracic circumference and chest wall movement,^14^ may have led to different RDI measurements compared to Caucasian populations. Therefore, we also suggest that the algorithm may require refinement for Asian populations or consideration of a different threshold cut‐off value in this demographic.

A recent meta‐analysis by Messaoud et al.^10^ indicated that the sensitivity of cardiac implants for sleep‐disordered breathing diagnosis ranged from 60 to 100%, with specificity from 50% to 100%. However, this study encompassed all CIED types, including PPMs, CRTs, and ICDs, with the predominant percentage being patients meeting indications for PPM implantation rather than heart failure patients requiring ICDs (only 12.2% are in ICD patients). In contrast, our study primarily focused on distinguishing between *severe* versus nonsevere sleep‐disordered breathing in patients requiring ICDs. This clinical emphasis is pertinent as patients who have untreated severe SDB have a poorer prognosis.

### 4.4 Utility of ESS and STOP‐Bang in patients with cardiomyopathy

In our population with cardiomyopathy, the ESS demonstrated poor sensitivity in detecting SDB (sensitivity = 17.6%). This could be attributed to the fact that patients with heart failure and cardiomyopathy often report less subjective daytime sleepiness, rendering the ESS less sensitive.

Conversely, the STOP‐Bang questionnaire exhibited high sensitivity but poor specificity (sensitivity = 94.1%, specificity = 0%). This discrepancy may arise from the comprehensive nature of the STOP‐Bang questionnaire, which considers both subjective experiences of tiredness and objective measurements including BMI, age, neck circumference, and gender.^12^ This is consistent with literature reporting high sensitivity in detecting OSA, particularly for moderate and severe cases (93% and 100% sensitivity, respectively). A comparison of the various screening tools is outlined in Table 2.

Considering the limitations inherent in each screening questionnaire (Table 2), it would be prudent for clinicians to integrate the results from all available tools. The ESS and STOP‐Bang questionnaires offer simplicity, cost‐effectiveness, rapid administration, and noninvasiveness. Additionally, for patients who already have an Implantable Cardioverter Defibrillator (ICD) implanted due to American College of Cardiology (ACC) indications for primary or secondary prevention, obtaining Respiratory Disturbance Index (RDI) measurements through remote monitoring is easily feasible. Therefore, there are no significant disadvantages or logistical difficulties associated with these measurements. Given the absence of a perfect noninvasive screening tool currently available, leveraging all existing tools can be highly beneficial and is strongly recommended for comprehensive assessment and management of SDB.

## 5 LIMITATIONS

A primary limitation of our study is the small cohort size, initially comprising 24 recruited patients, with only 18 completing the PSG study. Consequently, the observed prevalence of SDB, including obstructive, central, and mixed types, among our patient cohort meeting criteria for ICD implantation was 100%, which may not accurately reflect the prevalence in the general population. Additionally, this study was conducted at a single center, potentially limiting the generalizability of our findings. To address these limitations, we plan to expand our investigation with a larger patient cohort across multiple centers to validate our observations.

Furthermore, given the small cohort size of this pilot study, we included patients with obstructive sleep apnea (OSA), central sleep apnea (CSA), and mixed sleep apnea (MSA). OSA is characterized by pauses in nasal airflow with continuous respiratory effort, whereas CSA involves pauses in both nasal airflow and respiratory effort. MSA exhibits characteristics of both OSA and CSA. While we acknowledge the importance of these distinctions, we chose not to exclude CSA or MSA patients despite the different underlying mechanisms, as our primary aim was to gather preliminary data and assess the feasibility of the study across a spectrum of sleep apnea types.

In addition, current pacemaker algorithms are not sophisticated enough to differentiate between obstructive and central events due to their inability to distinguish cessation of thoracic or abdominal movement. Therefore, these algorithms cannot currently reveal the differences between OSA and CSA events.^7^ Unfortunately, this limitation is prevalent in existing impedance‐based algorithms and has been underscored by various studies,^1^, ^8^, ^10^ indicating the need for further technological advancements to aid in distinguishing central versus obstructive SDB.

The primary function of the pacemaker algorithms, as applied in our study, is not intended to substitute for PSG but rather to aid in identifying and screening for severe SDB in a population where SDB is often underdiagnosed. This approach allows for a broader understanding and detection of SDB, facilitating timely intervention and management.

We hope that future advancements in technology will enable more precise differentiation of sleep apnea types, enhancing the accuracy and utility of non‐PSG screening tools.

## 6 CONCLUSION

In conclusion, our findings underscore a high prevalence of SDB among patients with heart failure compared to the general population. However, conventional screening tools such as the Epworth Sleepiness Scale and STOP‐Bang questionnaires exhibit limited sensitivity and specificity, thus posing challenges in effectively identifying sleep‐disordered breathing in this cohort of patients with heart failure and cardiomyopathy.

Transthoracic impedance sensing with an advanced inbuilt algorithm emerges as a promising approach for screening of SDB and has identified a separate threshold for detecting severe SDB among patients with heart failure and cardiomyopathy. This study represents one of the earliest validations of the algorithm in an exclusively multiethnic Asian population with heart failure, marking a significant contribution to the field.

## FUNDING INFORMATION

This study was funded by Boston Scientific as an unrestricted grant.

## CONFLICT OF INTEREST STATEMENT

The authors declare that there are no conflicts of interest regarding the publication of this paper. All authors have disclosed any potential conflicts of interest, including financial, personal, or other relationships with other people or organizations that could inappropriately influence, or be perceived to influence, their work.

## DISCLOSURES

All authors have approved this manuscript for publication and confirm that this manuscript has not been published or submitted for publication elsewhere.

## ETHICS STATEMENT

This study was conducted in accordance with the principles outlined in the Declaration of Helsinki. Ethical approval was obtained from SingHealth Ethics Committee (Approval Number 2020/2141). All participants provided written informed consent prior to their inclusion in the study.

## Supporting information


Data S1.

## Data Availability

The original contributions presented in the study are included in the article/supplementary material, further inquiries can be directed to the corresponding author/s.
