# Supplementary material for: Utility of cardiac implantable electronic device algorithm for detecting severe sleep‐disordered breathing in cardiomyopathy
Source: J Arrhythm. 2024 Oct 8;40(6):1452–9. doi: 10.1002/joa3.13156 (PMC11632263; doi:10.1002/joa3.13156)
Supplement: Supplementary file 1 — Data S1. [file JOA3-40-1452-s001.docx]

Supplementary Material

**Supplementary Table 1: Measurements performed during Polysomnogram (PSG) study**

Sample table of the measurements taken during PSG

| **Measurements taken** | **Value** |
| --- | --- |
| Total AHI |  |
| REM AHI |  |
| NREM AHI |  |
| 3% ODI |  |
| % Total Sleep Time SpO2< 90% |  |
| Sleep Efficiency % |  |
| Sleep Onset Latency |  |
| N1 Sleep% |  |
| N2 Sleep% |  |
| REM Sleep% |  |
| N3 Sleep% |  |
| Arousal index |  |
| Index (#/hr) – Obstructive |  |
| Index (#/hr) – Mixed |  |
| Index (#/hr) – Central |  |
| Index (#/hr) – Total apnea |  |
| Index (#/hr) – Hypopnea |  |
| **Final diagnosis** |  |

**Supplementary Table 2: Sample Epworth Sleepiness Scale (ESS) questionnaire**

Patients were asked to complete the ESS and Stop-BANG questionnaires at first visit.

Patients were asked to rank the likelihood of dozing off in these instances.

| **Circumstance** | Would never dose (0) | Slight chance of dosing (1) | Moderate chance of dosing (2) | High chance of dosing (3) |
| --- | --- | --- | --- | --- |
| Sitting and reading |  |  |  |  |
| Watching TV |  |  |  |  |
| Sitting,inactive in a public place (eg. theater, cinema or meeting) |  |  |  |  |
| As a passenger in a car for an hour without a break |  |  |  |  |
| Lying down to rest in the afternoon when circumstances permits |  |  |  |  |
| Sitting and talking with someone |  |  |  |  |
| Sitting quietly after a lunch without alcohol |  |  |  |  |
| In a car, while stopped a few minutes in traffic(i.e, because of heavy traffic, traffic jam or red light |  |  |  |  |

**Supplementary Table 3: Sample Stop-BANG questionnaire**

Patients were asked to complete the ESS and Stop-BANG questionnaires at first visit.

| **STOP** | No (0) | Yes (1) |
| --- | --- | --- |
| Do you SNORE loudly (louder than talking or loud enough to be heard through closed doors)? |  |  |
| Do you often feel TIRED, fatigued, or sleepy during daytime? |  |  |
| Has anyone OBSERVED you stop breathing during your sleep? |  |  |
| Do you have or are you being treated for high blood PRESSURE? |  |  |
| **BANG** |  |  |
| BMI more than 35kg/m2? Age Over 50 year old? Neck circumference>16 inches (40cm)? Gender: Male? |  |  |
| BMI more than 35kg/m2? Age Over 50 year old? Neck circumference>16 inches (40cm)? Gender: Male? |  |  |
| BMI more than 35kg/m2? Age Over 50 year old? Neck circumference>16 inches (40cm)? Gender: Male? |  |  |
| BMI more than 35kg/m2? Age Over 50 year old? Neck circumference>16 inches (40cm)? Gender: Male? |  |  |
| **TOTAL SCORE** |  | |
